# Thermal Polymorphism in CsCB_11_H_12_

**DOI:** 10.3390/molecules28052296

**Published:** 2023-03-01

**Authors:** Radovan Černý, Matteo Brighi, Hui Wu, Wei Zhou, Mirjana Dimitrievska, Fabrizio Murgia, Valerio Gulino, Petra E. de Jongh, Benjamin A. Trump, Terrence J. Udovic

**Affiliations:** 1 Laboratory of Crystallography, Department of Quantum Matter Physics, University of Geneva, Quai Ernest-Ansermet 24, CH-1211 Geneva, Switzerland; 2 NIST Center for Neutron Research, National Institute of Standards and Technology, Gaithersburg, MD 20899-6102, USA; 3 National Renewable Energy Laboratory, Golden, CO 80401, USA; 4 Transport at Nanoscale Interfaces Laboratory, Swiss Federal Laboratories for Material Science and Technology (EMPA), Ueberlandstrasse 129, CH-8600 Duebendorf, Switzerland; 5 Materials Chemistry and Catalysis, Debye Institute for Nanomaterials Science, Utrecht University, 3584 CG Utrecht, The Netherlands; 6 Department of Materials Science and Engineering, University of Maryland, College Park, MD 20742, USA

**Keywords:** monocarba-hydridoborate, polymorphism, crystal structure, anion dynamics

## Abstract

Thermal polymorphism in the alkali-metal salts incorporating the icosohedral monocarba-hydridoborate anion, CB_11_H_12_^−^, results in intriguing dynamical properties leading to superionic conductivity for the lightest alkali-metal analogues, LiCB_11_H_12_ and NaCB_11_H_12_. As such, these two have been the focus of most recent CB_11_H_12_^−^ related studies, with less attention paid to the heavier alkali-metal salts, such as CsCB_11_H_12_. Nonetheless, it is of fundamental importance to compare the nature of the structural arrangements and interactions across the entire alkali-metal series. Thermal polymorphism in CsCB_11_H_12_ was investigated using a combination of techniques: X-ray powder diffraction; differential scanning calorimetry; Raman, infrared, and neutron spectroscopies; and ab initio calculations. The unexpected temperature-dependent structural behavior of anhydrous CsCB_11_H_12_ can be potentially justified assuming the existence of two polymorphs with similar free energies at room temperature: (*i*) a previously reported, ordered *R*3 polymorph stabilized upon drying and transforming first to *R*3*c* symmetry near 313 K and then to a similarly packed but disordered *I*43*d* polymorph near 353 K and (*ii*) a disordered *Fm*3 polymorph that initially appears from the disordered *I*43*d* polymorph near 513 K along with another disordered high-temperature *P*6_3_*mc* polymorph. Quasielastic neutron scattering results indicate that the CB_11_H_12_^−^ anions in the disordered phase at 560 K are undergoing isotropic rotational diffusion, with a jump correlation frequency [1.19(9) × 10^11^ s^−1^] in line with those for the lighter-metal analogues.

## 1. Introduction

Icosahedral hydridoborates M^x+^(B_12_H_12_)_x/2_ and their C-derivatives M^x+^(CB_11_H_12_)_x_ are extensively used in organic syntheses, medicine, nanoscale engineering, catalysis, metal recovery from radioactive waste, and recently as solid ionic conductors [1,2,3]. Their crystal structures are classified among so-called plastic (rotatory) crystals [4] as they show with temperature an order/disorder transition of dynamic nature into a state with orientationally disordered icosahedral anions B_12_H_12_^2−^ or CB_11_H_12_^−^. As shown by solid-state NMR, quasielastic neutron scattering (QENS) experiments and ab-initio calculations, the icosahedral anions undergo discrete symmetry-preserving reorientational jumps, even in their ordered state [5]. Upon transformation to the disordered state, the anion reorientational mobilities typically increase by several orders of magnitude, and the motions become more rotationally diffusive. While monocarba-hydridoborates of smaller alkali metals, such as Li and Na have been characterized in detail due to their potential importance as solid ionic conductors [2,6], the thermal polymorphism in KCB_11_H_12_ has been studied only recently [7]. No reports are available for RbCB_11_H_12_. Aside from the recent detailed room-temperature (*rt*) structural study of CsCB_11_H_12_ [8], only one study of thermal polymorphism in CsCB_11_H_12_ has been published, which was 18 years ago [9,10]. We have been motivated to understand more thoroughly the thermal polymorphism in CsCB_11_H_12_ in comparison with the monocarba-hydridoborate salts of the lighter alkali metals to get a broader insight into the nature of the anion–anion interaction and its effect on cation mobility. We will show that an understanding of the polymorphism in this compound is complicated by the presence of hydrated and metastable phases.

## 2. Results

### 2.1. Crystal Structures and Thermal Polymorphism

Structural characterizations were performed for the single-cation sample CsCB_11_H_12_, as well as for the Rb-doped Cs_0.93_Rb_0.07_CB_11_H_12_ salt.

#### 2.1.1. CsCB_11_H_12_

All measured SR-XPD data (two typical temperature-dependent SR-XPD patterns are shown in Figures S1 and S2) show the presence of an anhydrous polymorph γ observed at *rt* and three other polymorphs (β, α, and α′) observed on heating. The temperature region where the diffraction peaks of the polymorphs were observed depends on the heating/cooling rate. The list of the observed polymorphs is given in Table 1. The *γ*-polymorph has been characterized using single-crystal X-ray diffraction in refs. [9,10] as a hydrated phase with the composition CsCB_11_H_12_·1/3 H_2_O. A recent study of anhydrous CsCB_11_H_12_ has also been performed using single-crystal X-ray diffraction [8]. The thermal stability of the various polymorphs is analyzed in the Discussion.

**Figure 1:**
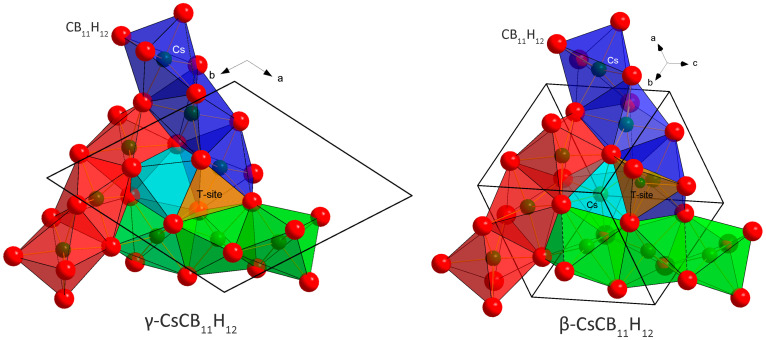
Comparison of the basic structural motif in the γ- (*R*3*c*) and β- (*I*43*d*) polymorphs of CsCB_11_H_12_. The crystal structures in both polymorphs can be constructed from three columns built from face-sharing Cs(CB_11_H_12_)_6_ octahedra (CB_11_H_12_^−^ anions simplified as red spheres) and inter-connected by edges (red, blue, and green columns, respectively). The three columns then share triangular faces with the fourth column (light blue). In the γ-polymorph, this column is empty, but may be occupied by a water molecule in the Cs/H_2_O ratio of 3/1. In the β-polymorph, Cs is randomly distributed inside all four columns with an occupancy of 0.75.

The γ-polymorph changes its symmetry reversibly from *R*3 to *R*3*c* at 313 K. The structural change is very subtle and practically invisible in the DSC curves (Figure S3) but clearly detected in diffraction patterns (Figures S1, S2, and S4). It is understood as a degeneration of two independent CB_11_H_12_^−^ anions in the asymmetric unit of *s.g. R3* into one in *s.g. R*3*c*, as also discussed in ref. [8]. The γ-polymorph of CsCB_11_H_12_ is stable down to at least 100 K as observed using low-temperature (*lt*) SR-XPD. Its hydrated version, studied in [9], contains 1/3 of a water molecule/*f.u.* disordered in the channels running along the *c*-axis shown in Figure 1 in light blue. In the anhydrous sample, the channel is empty with the C-H bond pointing inside it [8].

At 353 K, the γ-polymorph transforms into the β-polymorph. It has the symmetry of the cubic space group *I*43*d* and an unusual structural prototype of anti-Th_3_P_4_ [11] with Cs occupying 3/4 of the P positions, and CB_11_H_12_ localized on the Th positions. Its diffraction pattern corresponds to the phase called β in [9] where the water molecules (non-dried sample) are probably disordered on the remaining 1/4 of the P positions in the anti-Th_3_P_4_ prototype shown in light blue in Figure 1.

The anion packing in the γ- and β-polymorphs is very similar. In both structures, the anion–anion coordination number is CN = 12, but the packing is not a close packing (*ccp* or *hcp*). For *n* anions, the packing in both structures contains 4/3 *n* of octahedral O-sites and *n* tetrahedral T-sites. The distribution of O-sites is best described by three columns built from face-sharing Cs(CB_11_H_12_)_6_ octahedra and inter-connected by edges (red, blue, and green columns in Figure 1). The three columns then share octahedral faces with the fourth column (light blue). The size of shared triangular faces between the octahedra (O-O bottleneck) and between the octahedra and tetrahedra (O-T bottleneck) is similar and large enough (4.425 Å) in both structures to allow for cation mobility, which may be blocked by the presence of water molecules in hydrated samples. The Rietveld refinements suggest that the β-polymorph is a disordered version of γ with respect to both Cs distribution and anion rotational dynamics.

At 512–515 K, the diffraction peaks of β-polymorph disappear and peaks of α- and α′-polymorphs appear simultaneously, the latter disappearing at 533–598 K, depending on the heating rate. The α-polymorph is observed in diffraction data up to 623 K. Additional DSC-TGA curves measured up to 868 K (not shown here) indicate that the compound starts to lose mass (i.e., decomposes) noticeably by ~798 K. 

**Figure 2:**
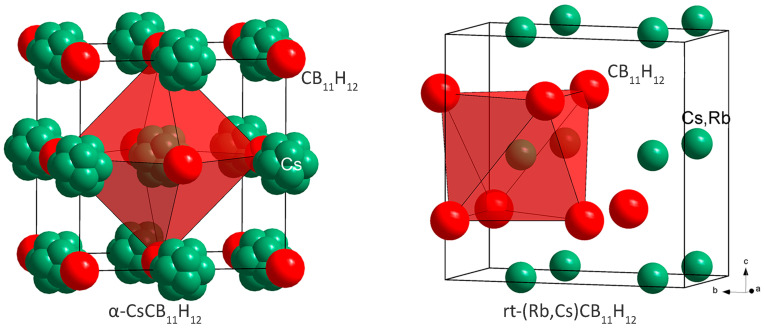
Structural representations of α-CsCB_11_H_12_ with anions *ccp*, and of mixed cation phase *rt*-(Rb,Cs)CB_11_H_12_ with anions hcp. The CB_11_H_12_^−^ anions are simplified as red spheres.

The α-polymorph is built as a cubic close packing (*ccp*) of anions with NaCl structure type, and its crystal structure can be refined in *Fm*3*m* or *Fm*3 symmetry without significant difference in the agreement factors (Figure 2). The powder pattern shown in Figure 4b of ref. [9] and attributed to a phase called α, corresponds in reality to a mixture of β- and α-polymorphs. The symmetry of the α′-polymorph is hexagonal (*P*6_3_*mc*). It was not possible to refine its crystal structure, due to low data quality. The labeling as α- and α′-polymorphs is not unambiguous because they appear simultaneously at the same temperature, but the α′-polymorph exists only in a limited temperature range. They may correspond to two polymorphs with very close free energies, as has been recently observed in CaB_10_H_10_ [12]. 

#### 2.1.2. Cs_0.93_Rb_0.07_CB_11_H_12_

In the *rt* diffraction data of a CsCB_11_H_12_ sample containing 7% Rb alkali-metal substitution, we have observed, besides the dominant peaks of γ-CsCB_11_H_12_, two other sets of diffraction peaks. The first one corresponds probably to the pure RbCB_11_H_12_ phase and disappears very rapidly. The second set has been successfully indexed on an orthorhombic cell and the structure solved in the space group *Pbcm* with the structural prototype of the high-pressure polymorph of NaOH, i.e., with anions *hcp* (Figure 2). As the fraction of this phase in the sample was very low (see Figure S9), the Rb/Cs ratio in this phase was not possible to refine. 

The Rietveld plots of all refined structures are given in the Supplementary Materials (Figures S5–S9). The CIF files may be obtained from the Fachinformationszentrum Karlsruhe, 76344 Eggenstein-Leopoldshafen (Germany), quoting the depository numbers CSD-2169329-216934.

### 2.2. Anion Dynamics

**Figure 3:**
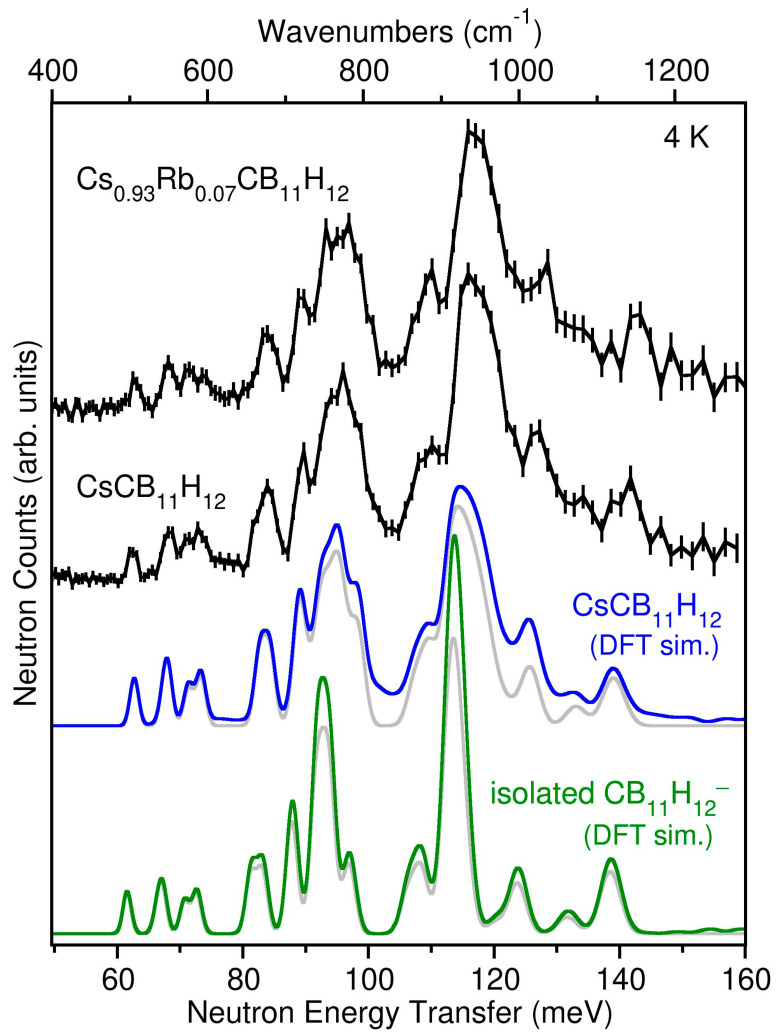
Neutron vibrational spectra (black) of CsCB_11_H_12_ and Cs_0.93_Rb_0.07_CB_11_H_12_ at 4 K compared to the simulated one + two-phonon densities of states from DFT phonon calculations of the optimized γ-CsCB_11_H_12_ *R*3-structure (blue) and the isolated CB_11_H_12_^−^ anion (green, from ref. [2], using a 30 × 30 × 30 supercell and full *C*5_v_ molecular symmetry). The simulated one-phonon densities of states considering only the fundamental single-phonon modes are shown for comparison in grey. Vertical error bars represent ±1 σ. (N.B., 1 meV ≈ 8.0655 cm^−1^).

In addition to the crystallographic results, the vibrational dynamics of both CsCB_11_H_12_ and Cs_0.93_Rb_0.07_CB_11_H_12_ samples at 4 K were characterized using NVS. The neutron vibrational spectrum for CsCB_11_H_12_ at 4 K is compared in Figure 3 with the simulated phonon density of states (PDOS) based on the DFT-optimized γ-CsCB_11_H_12_ *R*3-structure determined from the single crystal diffraction results. Due to the overwhelming large neutron scattering cross-section for H atoms with respect to other elements, the spectrum in this energy region is dominated by the normal modes involving the various possible CB_11_H_12_^−^ anion deformations, all of which entail significant H-atom displacements. The measured spectral signature is typically sensitive to the particular crystal structure [13,14] and in this instance agrees well with the simulated PDOS for the trigonal arrangement of CB_11_H_12_^−^ anions and Cs^+^ cations, taking into account the additional minor contributions expected from two-phonon combination bands. The presence of CsCB_11_H_12_ lattice effects on the measured PDOS is made clearer by the discrepancies observed between the CsCB_11_H_12_ spectrum and that calculated for the isolated CB_11_H_12_^−^ anion in Figure 3. We note that the spectrum for the CB_11_H_12_^−^ anion deformations measured with the Cs_0.93_Rb_0.07_CB_11_H_12_ sample is nearly identical to that for CsCB_11_H_12_, as expected based on this mixed-cation salt’s dominant γ-CsCB_11_H_12_-like structure and the lack of any significant vibrational perturbations from the relatively low concentration of Rb atoms. Further information about the characters and energies of the different CsCB_11_H_12_ phonon modes contributing to the simulated PDOS for the ordered *R*3 structure can be found in the animation file in the Supplementary Materials [15].

Quasielastic neutron scattering measurements were performed only on the anhydrous Cs_0.93_Rb_0.07_CB_11_H_12_ sample, although we expect the observed anion reorientational behavior to largely mimic that of pure CsCB_11_H_12_. At 560 K, the CB_11_H_12_^−^ anions in the predominant disordered polymorph α displays a reorientational jump correlation frequency of 1.19(9) × 10^11^ s^−1^, a rather high reorientational mobility in general agreement with the values extrapolated from lower-temperature QENS data for the lighter-metal Li, Na, and K carba-hydridoborates [7,16] (see Figures S10 and S11). Moreover, this 560 K jump frequency is almost 20× higher than that of the B_12_H_12_^2−^ anions in the symmetry-related Cs_2_B_12_H_12_ [17,18], which is not unexpected since the B_12_H_12_^2−^ anions in Cs_2_B_12_H_12_ are relatively more “confined”, i.e., they are surrounded by twice as many cations as the CB_11_H_12_^−^ anions in the Rb-doped CsCB_11_H_12_ salt. The momentum transfer dependence of the elastic incoherent structure factor at 560 K (Figure S12) indicates that the CB_11_H_12_^−^ anion reorientations at this high temperature are more akin to isotropic rotational diffusion, a behavior not yet fully reached by the B_12_H_12_^2−^ anions in Cs_2_B_12_H_12_ (by 530 K) [18] or by the CB_11_H_12_^−^ anions in the Li, Na, and K carba-hydridoborates (by 473–480 K) [7,16].

We note that high CB_11_H_12_^−^ anion rotational mobility can also significantly affect the translational mobility of the cations [16]. Although the Cs^+^ cation diffusive mobility in disordered α-CsCB_11_H_12_ is certainly much lower than that for the lighter and smaller alkali-metal cations (Li^+^, Na^+^, and K^+^) in their analogous *ht*-disordered MCB_11_H_12_ phases [2,7], the emergence of rotationally fluidic anions for this phase can still provide a more accommodating potential-energy landscape [16] for greatly enhancing the cation conductivity compared to that for the lower-temperature ordered structure. Indeed, increased anion rotational mobility may be at least partially responsible for the four-orders-of-magnitude increase in Cs^+^ conductivity observed above ~483 K (σ = 5.5 × 10^−5^ S cm^−1^) for the *ht*-disordered α-polymorph of the related Cs dodecahydro-7,8-dicarba-*nido*-undecaborate salt, Cs-7,8-C_2_B_9_H_12_, as compared to its low- and medium-temperature ordered polymorphs [19], although the authors attributed this conductivity jump solely to favorable α-polymorph structural effects. 

## 3. Discussion

### 3.1. Metastable and Stable Polymorphs

Understanding of CsCB_11_H_12_ thermal polymorphism is complicated by hydrated pristine samples. Contrary to hydrated Cs_2_B_12_X_12_ (X = Cl, Br, and I) and alkali-metal salts A_2_B_12_F_12_ where the water molecule coordinates via its lone pair with the cations [20,21], in hydrated CsCB_11_H_12_, the disordered water molecule coordinates with the CB_11_H_12_^−^ anions. DFT structural optimization of *R*3 γ-CsCB_11_H_12_·1/3 H_2_O (with artificial H_2_O site ordering) points to the formation of di-hydrogen bonds between the hydrogens of the water molecule and hydrogens from two different CB_11_H_12_^−^ anions. As the water molecule is normally disordered in the channels, we were unable to get a more precise answer. The single crystal results from ref. [8] suggest the C-H bond to point inside the channels, i.e., towards the possible water molecules, and DFT calculations confirm that this particular anion orientation is energetically preferred. 

The drying procedure of the as-purchased hydrated samples under dynamic vacuum at 593–693 K provides anhydrous powders verified using Raman and IR spectroscopies (Figures S13 and S14) where the characteristic broad O-H stretching and H-O-H bending bands are missing at 3200–3550 and 1600 cm^−1^, respectively. 

**Figure 4:**
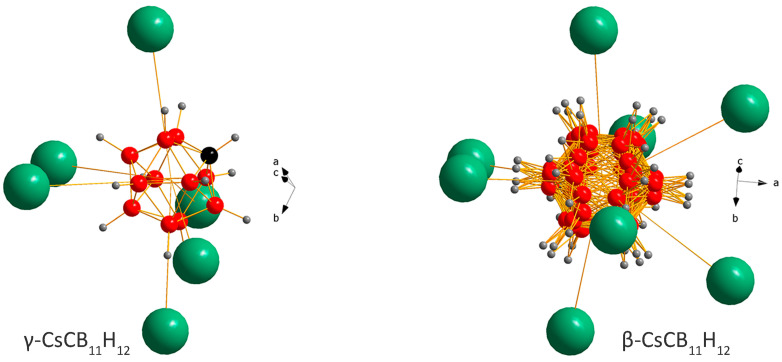
Coordination of the CB_11_H_12_^−^ anion in the crystal structures of γ- and β-CsCB_11_H_12_. The γ-structure as obtained from single-crystal data [8] is optimized here with DFT also allowing carbon localization. While the anion is ordered in the former, it is rotationally disordered in the latter. The water molecules in hydrated phases occupy the channels on the right side from the anion in γ and share the positions with Cs atoms in the ratio 1:3 in β.

CsCB_11_H_12_ behaves unexpectedly with temperature and its polymorphism can be understood only by accepting similar *rt* free energies of the ordered γ- and disordered α-polymorphs. One possible scenario for CsCB_11_H_12_ is the metastability of ordered γ- and stability of disordered α-polymorphs at *rt*. In such a scenario, the as-purchased sample contains hydrated γ-phase. When dried, the channels occupied by water molecules (light blue in Figure 1) become empty, but the CsCB_11_H_12_ framework stays intact on fast cooling from the drying temperature to *rt*. The coordination of the CB_11_H_12_^−^ polyanion in the γ-phase with *R*3/*R*3c symmetry points to a metastable structure (Figure 4 left) called as unexpected in ref. [8]. Without the water molecules, which were located in the channels on the right side from the anion in Figure 4 left, the Coulombic forces between Cs^+^ and CB_11_H_12_^−^, even if balanced according to DFT calculations, can be easily pushed out of balance. On the other hand, the forces seem to be better balanced in the disordered β-polymorph (Figure 4 right). In the temperature-dependent SR-XPD data where the heating has been stopped before the appearance of α- and α′-phases, the β-polymorph does not transform back to the γ-polymorph on cooling and persists in the sample down to 100 K. 

On heating, the γ-polymorph transforms to its disordered variant β, and at 512–515 K, the α- and α′-polymorphs crystallize from melted β. Please note the diffuse intensity signal starting from this temperature marking the presence of a melted sample fraction, which does not recrystallize into α- and α′-polymorphs due to the slow crystallization kinetics of the latter two polymorphs (Figures S1 and S2). The sample behavior on cooling depends on the cooling rate. During fast cooling (10 K/min) used in Figure S1 and during the fast DSC scans (Figure S3 left), the melted fraction of the sample does not crystallize as the stable α-polymorph, but rather as metastable β, which transforms upon further cooling into the γ-polymorph. The crystallization of β- and γ- instead of the *α*-polymorph on cooling can be explained by the Ostwald step rule [22] stating that the phase with the lower kinetic barrier forms first due to its faster crystallization kinetics. When slow cooling is used (2 K/min), Figure S2 does not show any recrystallization of β- and γ-polymorphs. The persisting diffuse scattering visible in both diffraction data sets, fast and slow heating/cooling rates, has its origin in the melted fraction of the sample, which does not transform into β- and γ-phases and stays glassy. In slow DSC scans (Figure S3 right), the β- and γ-recrystallization events are still visible on cooling, but with decreasing intensity compared to heating. The difference between the temperature-dependent SR-XPD data and the DSC curve may be understood as due to different crystallization kinetics between a closed system (capillary in the diffraction experiment) and an open system (sample holder in the DSC experiment). The two unexplained signals in the cooling part of DSC scans without an equivalent in the heating part are tentatively attributed to glassy transitions in the melted part of the sample.

We have suggested in the above discussion that the disordered α-polymorph may be the stable (i.e., thermodynamically preferred) structure at *rt*. Nonetheless, at first glance, it would seem that the γ-polymorph is the actual stable *rt*-form as it is supported by the crystallization of γ from the water solution in ref. [8]. Yet, we caution that this somewhat unexpected γ-crystallization may have been aided by the participation of water molecules in this case, a structural pathway which may not necessarily occur in an anhydrous crystallization environment.

### 3.2. Cation Coordination in Alkali-Metal Carba-Hydridoborates

**Figure 5:**
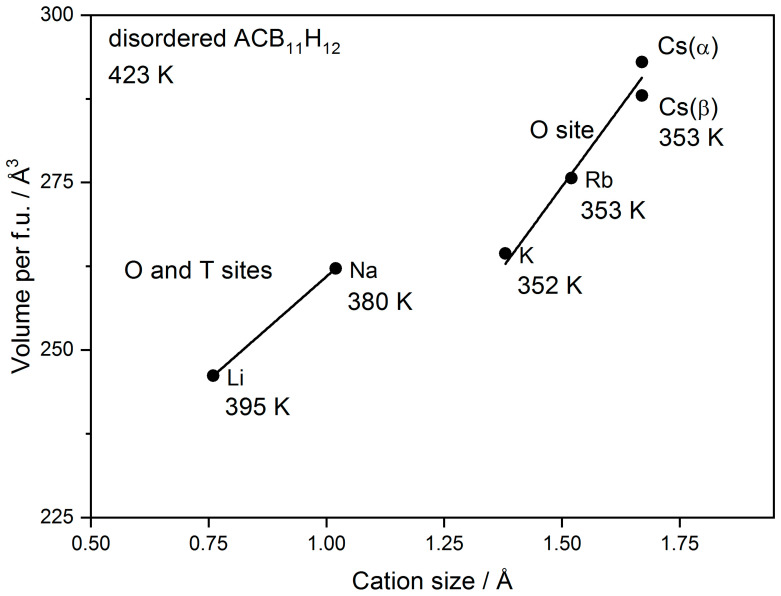
Dependence of volume per formula unit in ACB_11_H_12_ (A = Li, Na, K, Rb, and Cs) on alkali cation radius in octahedral coordination according to Shannon [23]. The temperature of cation- and anion-disordering is also given. The structure of all compounds is disordered with *ccp* or *hcp* anion packing with the exception of disordered β-CsCB_11_H_12_.

We will now discuss the relation of Cs and other alkali-metal carba-hydridoborates. In all *rt*-structures, the alkali metal is located in O-sites. In the ordered phases of Li, Na, and K [2,7], the cation is displaced from the center of the O-site towards a triangular face of the octahedron (CB_11_H_12_)_6_ due to the size effect. The Cs is disordered around the octahedron center in the α-polymorph. The localization of the relatively smaller Li and Na in O-sites compared to hydridoborates Li_2_B_12_H_12_ and Na_2_B_12_H_12_ where it occupies T-sites may be explained by the repulsion between the cation and the least electronegative H bonded to the C atom pointing preferentially towards the T-site in carba-hydridoborate as recently shown by molecular dynamics simulations [16]. In disordered *ht*-phases of Li and Na carba-hydridoborates, the cation occupies both O- and T-sites [2], but in K [7] and Rb (structural results not shown here, manuscript under preparation), carba-hydridoborates the cation occupies only the O-site. The difference in the occupation of O- and T-sites by the cation between disordered carba-hydridoborates of Li and Na on one side and K, Rb, and Cs on the other side is visible as a difference in the volume/*f.u.* vs. cation size plot shown in Figure 5. The temperature of cation and anion disordering is also given in the figure showing its dependence on the cation coordination. 

## 4. Materials and Methods

### 4.1. Samples Preparation

CsCB_11_H_12_ and Cs_0.93_Rb_0.07_CB_11_H_12_ were obtained from Katchem (The mention of all commercial suppliers in this paper is for clarity and does not imply the recommendation or endorsement of these suppliers by any involved institution). The as-purchased products were hydrated and were first superficially dried under a dynamic vacuum at 453 K for 12 h. Any remaining residual water was removed by evacuating at 603 K for 16 h in a quartz tube followed by the removal of the tube from the oven, resulting in a cooldown to *rt* over approximately 0.5 h.

### 4.2. Synchrotron Radiation X-ray Powder Diffraction (SR-XPD)

The data used for the crystal structure solution and refinement were collected at the Swiss Norwegian Beamlines of ESRF (European Synchrotron Radiation Facility, Grenoble, France) between 100 and 723 K. Temperature-dependent SR-XPD data were collected on the Dectris Pilatus M2 detector at wavelengths of 0.8212, 0.7399, and 0.64113 Å. The 2D images were integrated and treated with the locally written program Bubble. For all measurements, the samples were sealed into borosilicate capillaries of diameter 0.5 mm (under argon atmosphere), which were spun during data acquisition. The temperature above *rt* was controlled with a hot air blower calibrated with thermal dilatation of silver. The temperature below *rt* was controlled with a Cryostream 700 (Oxford Cryosystems, Long Hanborough, UK). The wavelength was calibrated with a NIST SRM640c Si standard. Additional SR-XPD patterns were collected for CsCB_11_H_12_ in a 1 mm diameter sealed quartz capillary (unspun) at the Advanced Photon Source at Argonne National Laboratory at a wavelength of 0.45246 Å using a two-dimensional amorphous Si-plate detector. The two-dimensional data were converted to one-dimensional data using GSAS-II [24]. Crystal structures were solved ab-initio using the software Fox [25] and refined with the Rietveld method using TOPAS [26] or GSAS [27]. The CB_11_H_12_^−^ anion was modelled as a rigid body with an ideal icosahedral shape and with B-H and B-B distances of 1.81 and 1.16 Å, respectively. As carbon localization is largely hindered by the low X-ray scattering contrast between B and C atoms, the anion was modelled as a B_12_H_12_ cluster. The interatomic distances and coordination polyhedra were analyzed using the program DIAMOND [28]. 

### 4.3. Differential Scanning Calorimetry (DSC)

Differential scanning calorimetry (DSC) measurements for CsCB_11_H_12_ were performed using a Mettler Toledo HP DSC 1-STAR (Mettler Toledo, Greifensee, Switzerland). Approximately 5−10 mg of the sample were loaded in 40 mg aluminum crucibles, closed with a lid inside a glove box, and transferred to the DSC under an inert atmosphere. The measurements were performed under an Ar flow, using heating and cooling ramps of 2 or 10 K/min in the desired temperature range.

### 4.4. Raman Spectroscopy

The Raman spectra at *rt* were collected using the spectrometer Horiba (Kyoto, Japan), LabRAM HR Evolution using the same samples in the capillary that were used in the SR-XPD experiments.

### 4.5. Fourier Transformation Infrared Spectroscopy (FTIR)

Infrared spectra were collected in diffuse reflectance infrared Fourier transform spectroscopy (DRIFTS) mode, using a PerkinElmer 2000 spectrometer (PerkinElmer, Waltham, MA, USA). Thirty-two scans were accumulated in the 4000−900 cm^−1^ range with a resolution of 4 cm^−1^. KBr powder was used to collect the background. To prevent air contamination, the sample was placed in an airtight sample holder, with KBr windows, inside the glove box.

### 4.6. Neutron Scattering Measurements

Neutron scattering measurements for CsCB_11_H_12_ were performed at the NIST Center for Neutron Research (NCNR) using thin flat-plate-shaped samples in reflection to minimize neutron beam attenuation from the highly-neutron-absorbing ^10^B present in natural boron. The H-weighted phonon density of states (PDOS) at 4 K was measured using neutron vibrational spectroscopy (NVS) on the Filter-Analyzer Neutron Spectrometer (FANS) [29] using the Cu(220) monochromator with pre- and post-collimations of 20’ of arc, yielding a full-width-at-half-maximum (FWHM) energy resolution of about 3% of the neutron energy transfer. Quasielastic neutron scattering (QENS) spectra were measured at 100 K and 560 K on the Disc Chopper Spectrometer (DCS) [30], utilizing incident neutron wavelengths of 4.8 Å and 8 Å with respective resolutions of 56 µeV and 30 µeV FWHM. The instrumental resolution function was obtained from the purely elastic 100 K QENS spectrum. All neutron data analyses were done with the DAVE software package [31]. Quantitative elemental analysis of the Cs_1-*x*_Rb*_x_*CB_11_H_12_ mixed cation alloy was also performed at the NCNR using the NGD Cold-Neutron Prompt Gamma-Ray Activation Analysis Spectrometer [32]. The Cs/Rb atomic ratio was determined after neutron irradiation of the sample from analysis of spectral peaks in the gamma-ray decay spectrum at the element-specific gamma-ray energies of 605 keV and 796 keV (for ^134^Cs) and 1077 keV (for ^86^Rb).

### 4.7. Density Functional Theory (DFT) Calculations

To complement the CsCB_11_H_12_ structural refinements and NVS measurement, first-principles calculations were performed within the plane-wave implementation of the generalized gradient approximation to Density Functional Theory (DFT) using a Vanderbilt-type ultrasoft potential with Perdew–Burke–Ernzerhof exchange-correlation and the program QUANTUM ESPRESSO [33]. A cutoff energy of 544 eV and a 2 × 2 × 2 k-point mesh (generated using the Monkhorst-Pack scheme) were used and found to be enough for the total energy to converge within 0.01 meV/atom. For comparison with the NVS measurement, the PDOS was calculated for the 0 K DFT-optimized CsCB_11_H_12_ structure (as determined using SR-XPD) using the supercell method with finite displacements [34,35] and was appropriately weighted to take into account the H, Cs, B, and C total neutron scattering cross sections.

## 5. Conclusions

The various structural and dynamical results presented above for CsCB_11_H_12_ suggest a more complex thermal polymorphic behavior than expected from purely thermodynamic considerations, with observed structural transformations intimately dependent on the specific thermal history. In particular, a likely similarity in free energies between metastable and stable polymorphs combined with the sensitivity to the magnitude of the thermal ramping and cooling rates on the preferred order-disorder and recrystallization pathways may result in kinetically preferred rather than thermodynamically preferred structural configurations. The situation is made more complicated by the potential presence of H_2_O molecules that must be removed from within the void spaces of the synthesized CsCB_11_H_12_ lattice, lest they adversely affect the preferred structural pathways of otherwise anhydrous CsCB_11_H_12_. Such trace H_2_O is strongly bound and/or kinetically trapped and can only be removed using extensive thermal evacuation near 600 K.

The anion reorientational mobility of the CB_11_H_12_^−^ anions in the disordered *Fm*3-polymorph at 560 K (>10^11^ jumps s^−1^) is found to be in line with the temperature-dependent trend reported for the other disordered, lighter-alkali-metal analogues. At this high temperature, QENS results indicate that the anions undergo a reorientational mechanism akin to isotropic rotational diffusion. This rotational-liquid-like mobility likely has some synergistic influence on enhancing the translational diffusive mobility of the Cs^+^ cations, such as typically observed for the cations in various other related disordered polyhedral hydridoborate and carba-hydridoborate salts.

It is still unclear which polymorph of CsCB_11_H_12_ is thermodynamically preferred at *rt*. The answer might be found by more extensive ab initio calculations comparing the relative stabilities of the *R*3- and *Fm*3-polymorphs, although the latter polymorph is already disordered at *rt*, making a definitive theoretical assessment of its relative stability more problematic. Finding a minimal-energy ordered version of this *ccp* structure that might appear at a lower temperature will require a nontrivial computational search of *ccp*-like structures starting from lower crystal symmetries. Of course, experimentally observing such a low-temperature structure would be the ultimate goal. As it would be both beneficial to this study and of fundamental interest to understand the structural trends across the entire alkali-metal family of MCB_11_H_12_ salts, we are currently extending our investigations to characterize the thermal polymorphism in the still largely unexplored RbCB_11_H_12_ analogue.

## Figures and Tables

**Table 1:** Thermal polymorphs observed in anhydrous CsCB_11_H_12_ with their space group symmetry (*s.g.*), lattice parameters, cell volume, and the number of formula units (*f.u.*)/unit cell (*Z*). The naming of the polymorphs is according to ref. [9].

Polymorph	*s.g.*	*a* [Å]	*c* [Å]	*V* [Å^3^]	*Z*	T_exp_ [K]
γ	*R*3	20.9533 (1)	13.2400 (1)	5034.1 (1)	18	303
		20.7818 (2)	13.0935 (2)	4897.3 (1)		156
β	*I*43*d*	15.1493 (6)		3476.8 (4)	12	423
α	*Fm* 3	10.6491 (9)		1207.7 (3)	4	523
α′	*P*6_3_*mc*	7.3987 (5)	12.5639 (3)	595.5 (2)	2	539

## Data Availability

All data supporting reported results may be obtained upon the request from the authors.

## Supplementary Materials

The following supporting information can be downloaded at: https://www.mdpi.com/article/10.3390/molecules28052296/s1: Figures S1 and S2: Temperature dependent powder patterns; Figure S3: DSC curves; Figure S4: The diffraction peak positions (blue lines) generated by R3 and R3c space groups compared to the respective 298 K and 353 K SR-XPD patterns measured for CsCB11H12; Figures S5–S9: Rietveld plots; Figure S10: QENS spectrum; Figure S11: Arrhenius plot of jump correlation frequency; Figure S12: Elastic incoherent structure factors as a function of *Q*, Figure S13: Raman spectrum of CsCB11H12 measured at room temperature; Figure S14: IR spectra of CsCB11H12 measured at room temperature, Phonon animation file.

## Sample Availability

Samples of the compounds are not available from the authors.
