# Supplementary material for: Thermal Polymorphism in CsCB11H12
Source: Molecules. 2023 Mar 1;28(5):2296. doi: 10.3390/molecules28052296 (PMC10005072; doi:10.3390/molecules28052296)
Supplement: Supplementary file 1 [file molecules-28-02296-s001.zip › molecules-2202613-SM.pdf]

Supporting Information

Thermal polymorphism in $CsCB_{11}H_{12}$

Radovan Černý, Matteo Brighi, Hui Wu, Wei Zhou, Mirjana Dimitrievska, Fabrizio Murgia, Valerio Gulino, Petra E. de Jongh, Benjamin A. Trump, Terrence J. Udovic

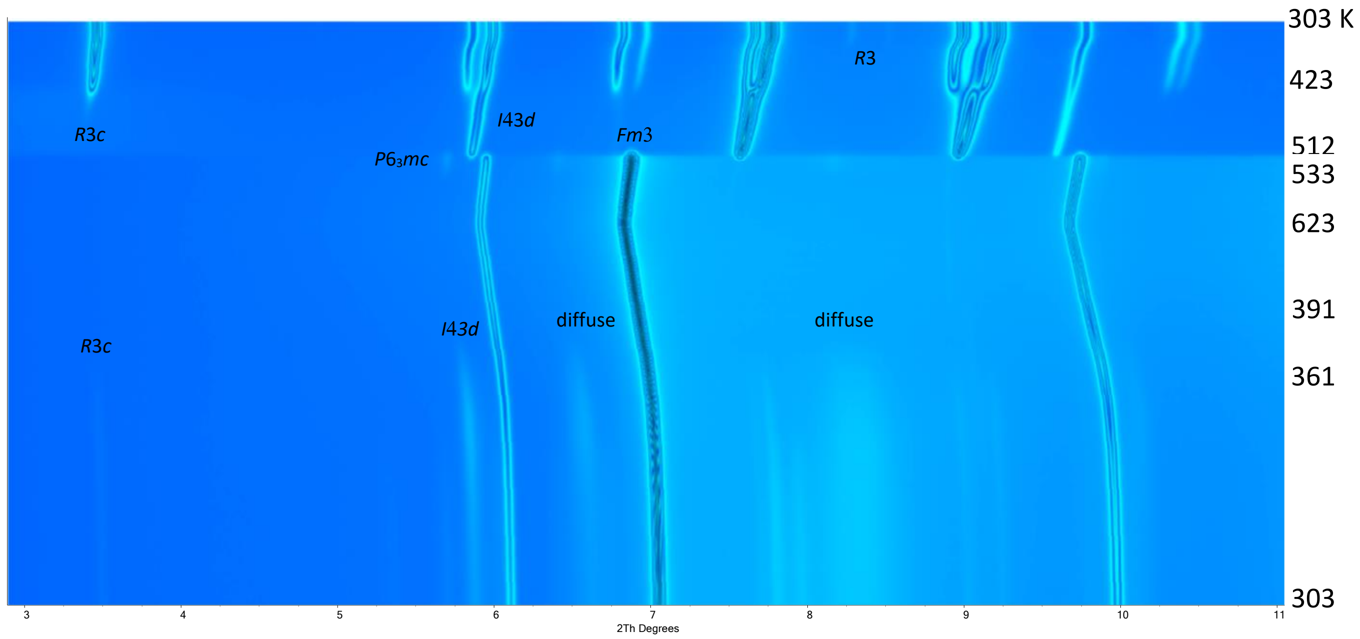

Figure S1: Temperature dependent SR-XPD data measured on dry $CsCB_{11}H_{12}$ sample with fast heating/cooling rate of 10 K/min, wavelength λ= 0.64113 Å. The peaks of different polymorphs are labelled with their space group symbols. Observed diffuse intensity is labelled too. Different colour modes have been chosen in Figures S1 and S2 to increase the contrast between Bragg peaks and diffuse intensity.

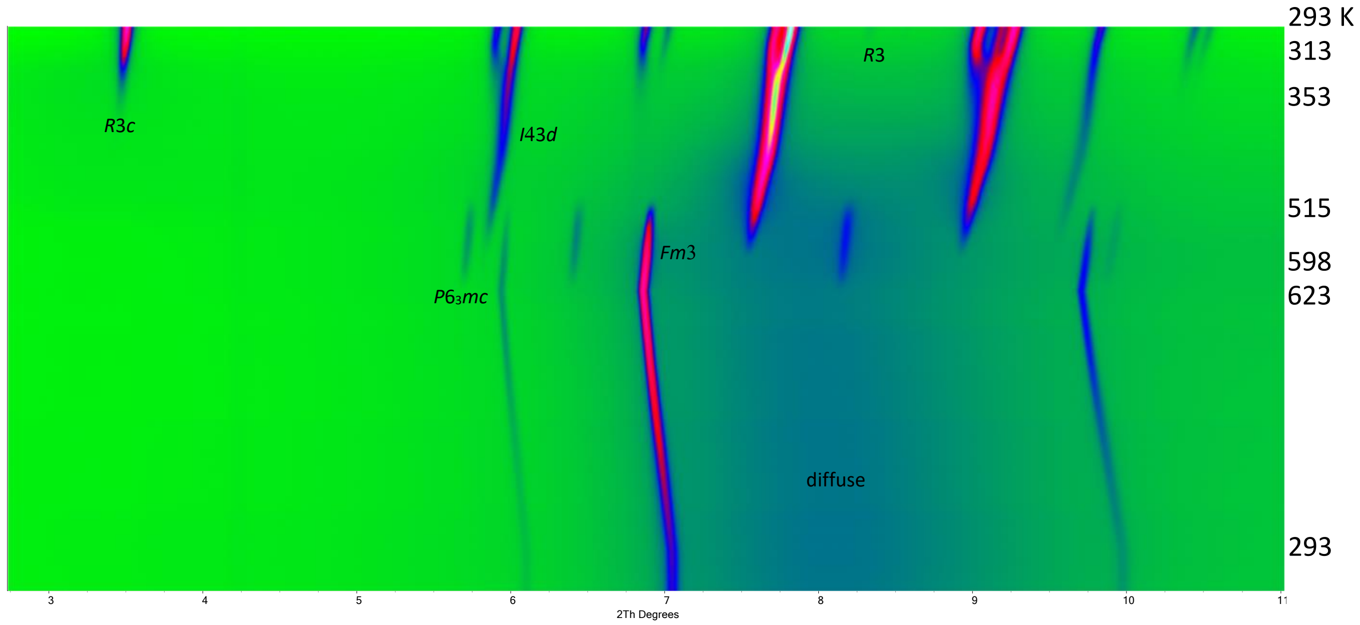

Figure S2: Temperature dependent SR-XPD data measured on dry $CsCB_{11}H_{12}$ sample with slow heating/cooling rate of 2 K/min, wavelength λ= 0.64113 Å. The peaks of different polymorphs are labelled with their space group symbols. Observed diffuse intensity is labelled too. Different colour modes have been chosen in Figures S1 and S2 to increase the contrast between Bragg peaks and diffuse intensity.

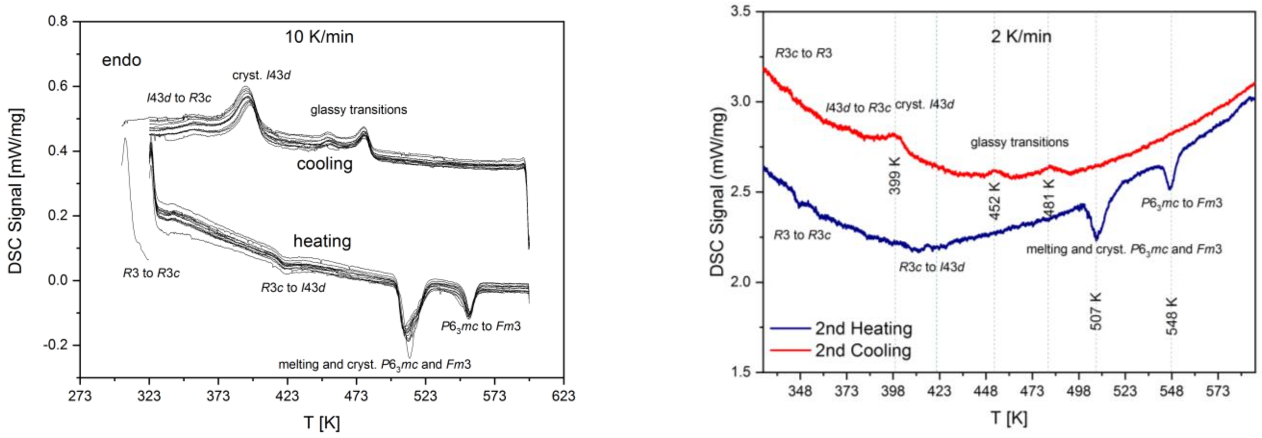

Figure S3: DSC curves of dry $CsCB_{11}H_{12}$. Fast heating/cooling rate (10 K/min) on the left, slow heating/cooling rate (2 K/min) on the right.

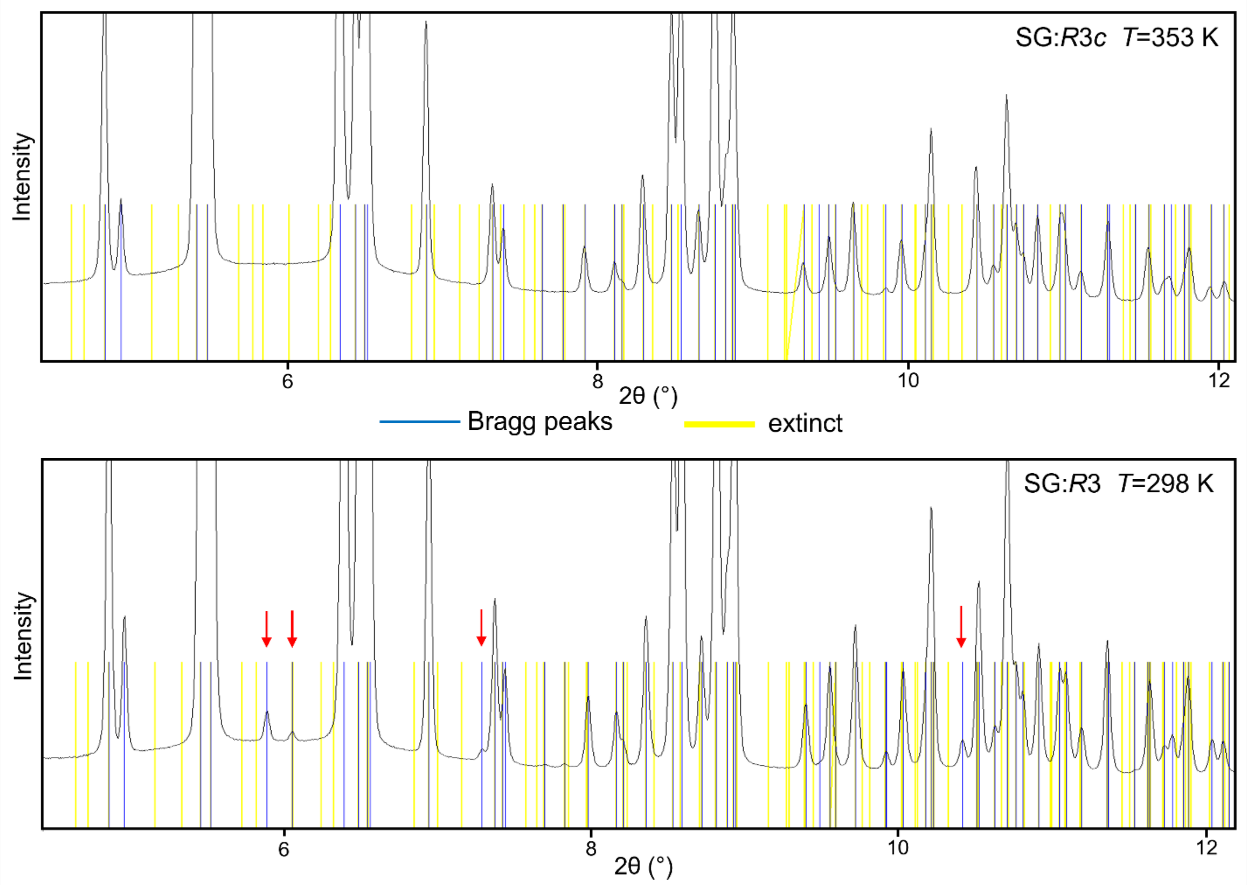

Figure S4: The diffraction peak positions (blue lines) generated by *R*3 and *R*3*c* space groups compared to the respective 298 K and 353 K SR-XPD patterns measured for $CsCB_{11}H_{12}$; wavelength λ= 0.45246 Å. Red arrows mark positions of Bragg peaks that are allowed for *R*3 symmetry and observed at 298 K, but are forbidden for *R*3*c* symmetry and absent at 353 K.

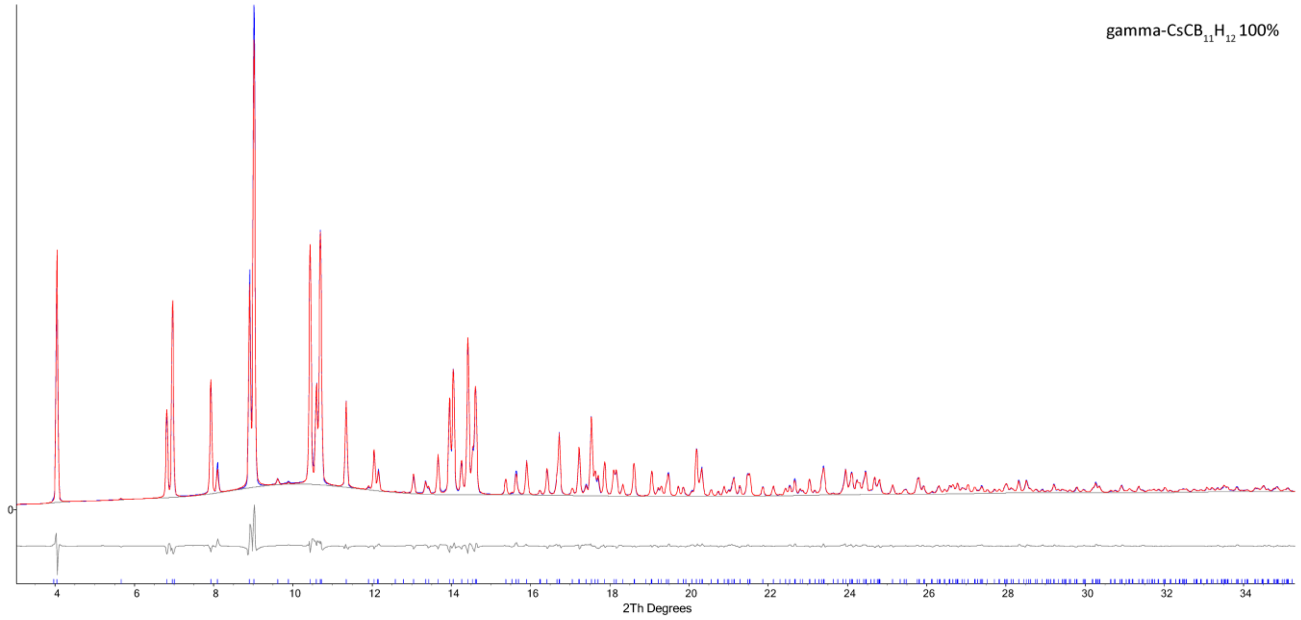

Figure S5: Rietveld plot for refinement of γ-$CsCB_{11}H_{12}$. Data from SNBL, T=303 K, λ=0.7399 Å, R_{wp}(corrected for background)=6.5 %, χ^2=14.

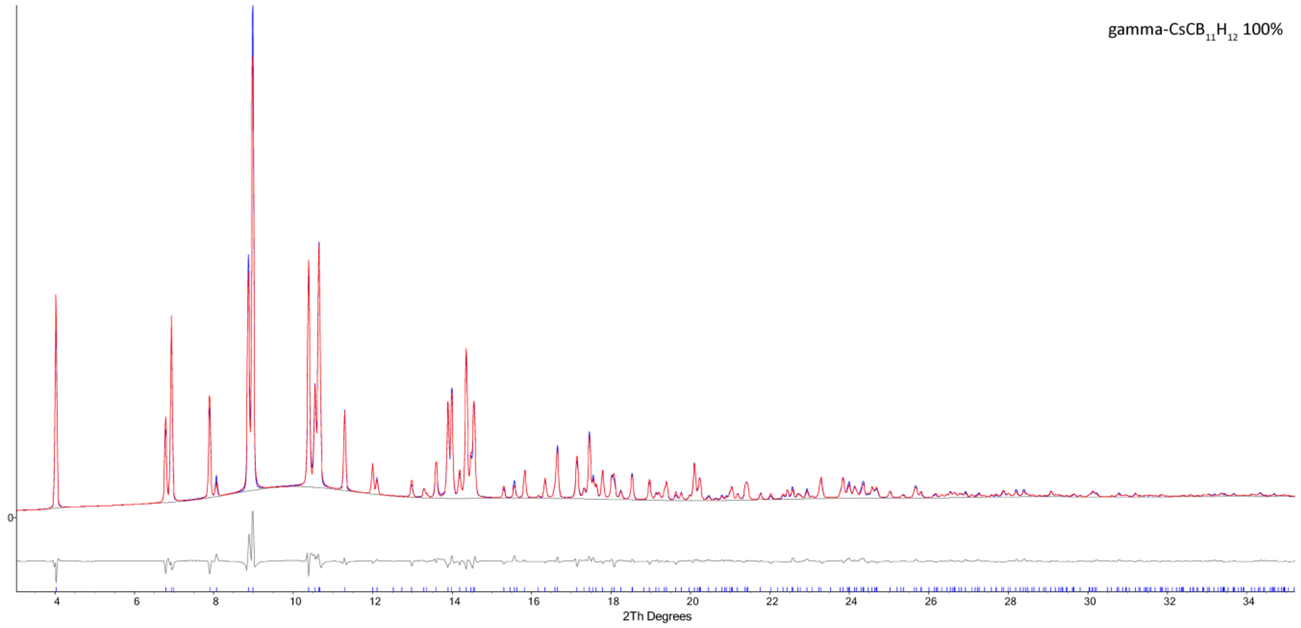

Figure S6: Rietveld plot for refinement of γ-$CsCB_{11}H_{12}$. Data from SNBL, T=333 K, λ=0.7399 Å, R_{wp}(corrected for background)=9.7 %, χ^2=17.

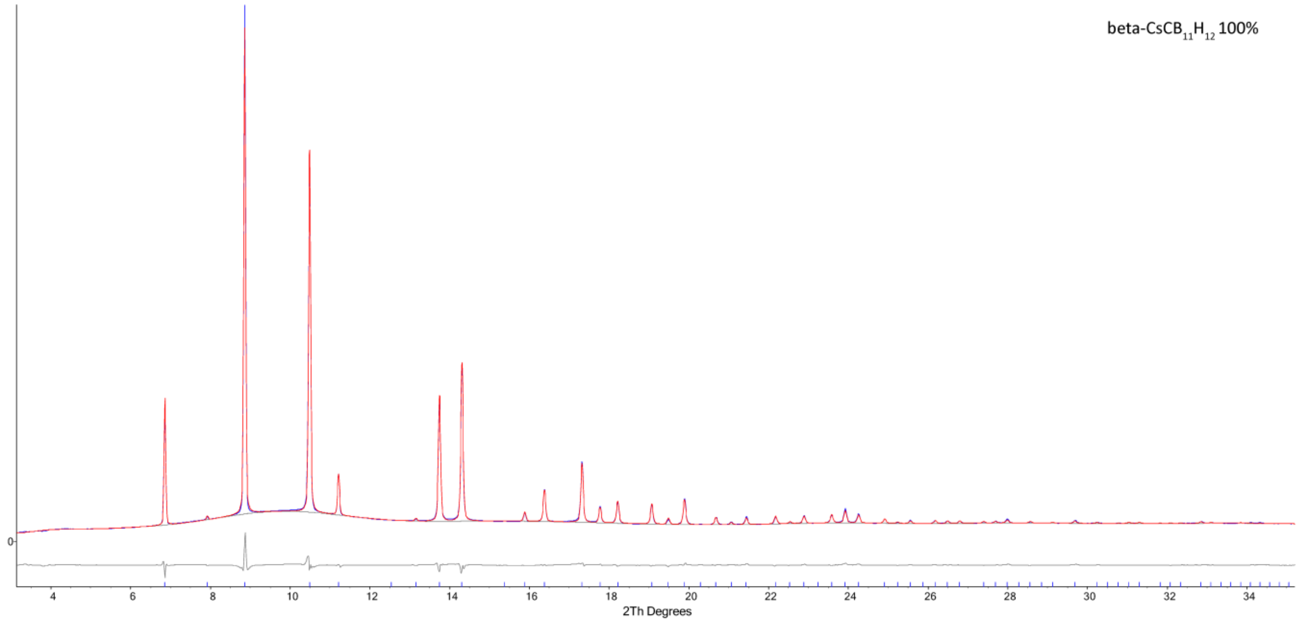

Figure S7: Rietveld plot for refinement of β-$CsCB_{11}H_{12}$. Data from SNBL, T=423 K, λ=0.7399 Å, R_{wp}(corrected for background)=7.3 %, χ^2=10.

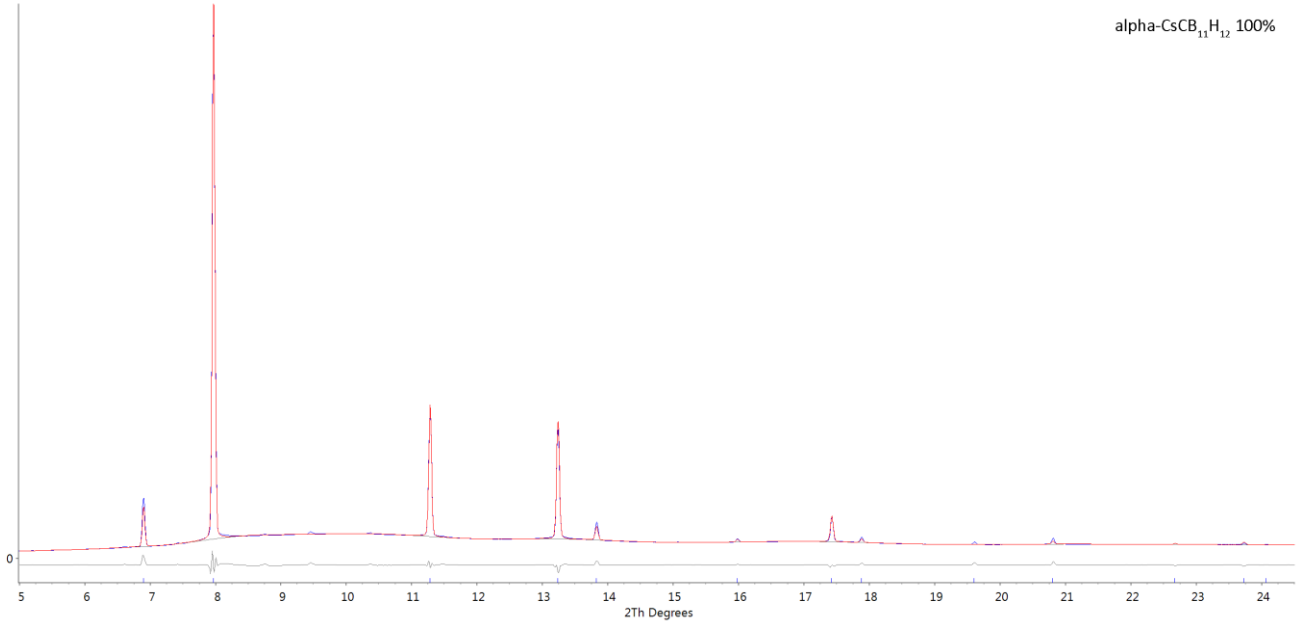

Figure S8: Rietveld plot for refinement of α-$CsCB_{11}H_{12}$. Data from SNBL, T=523 K, λ=0.7399 Å, R_{wp}(corrected for background)=8.6 %, χ^2=12.

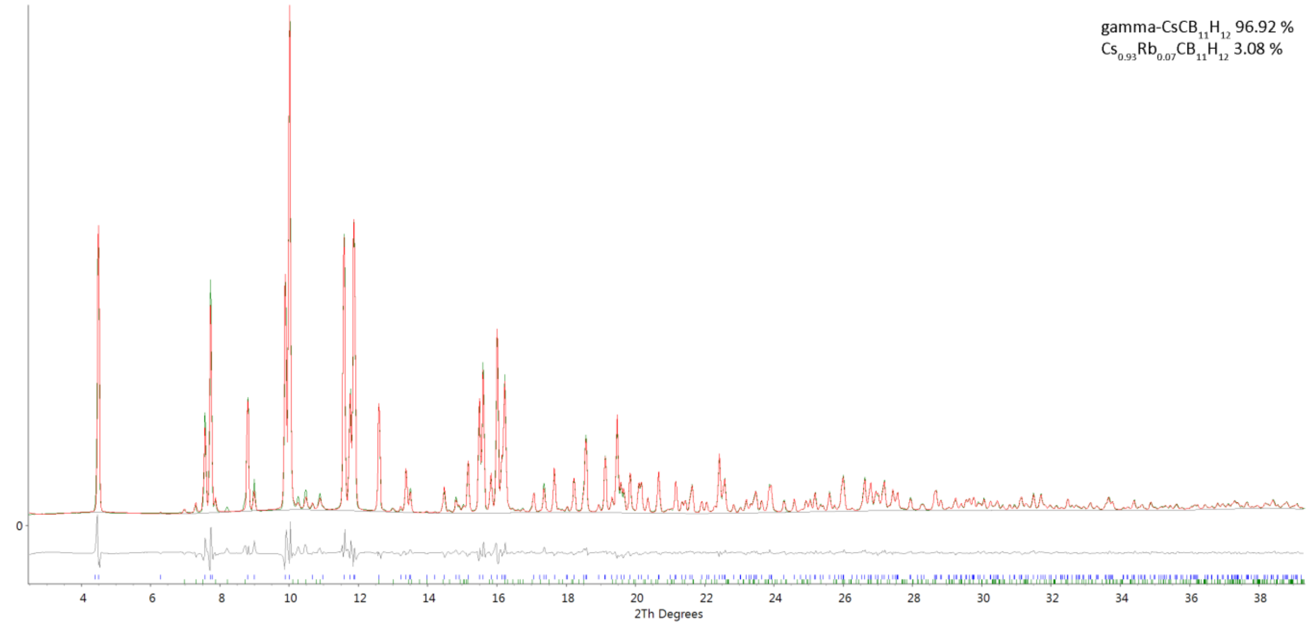

Figure S9: Rietveld plot for refinement of $Cs_{0.93}Rb_{0.07}CB_{11}H_{12}$. Data from SNBL, T= 298 K, λ=0.8212 Å, R_{wp}(corrected for background)= 9.7 %, χ^2= 28.

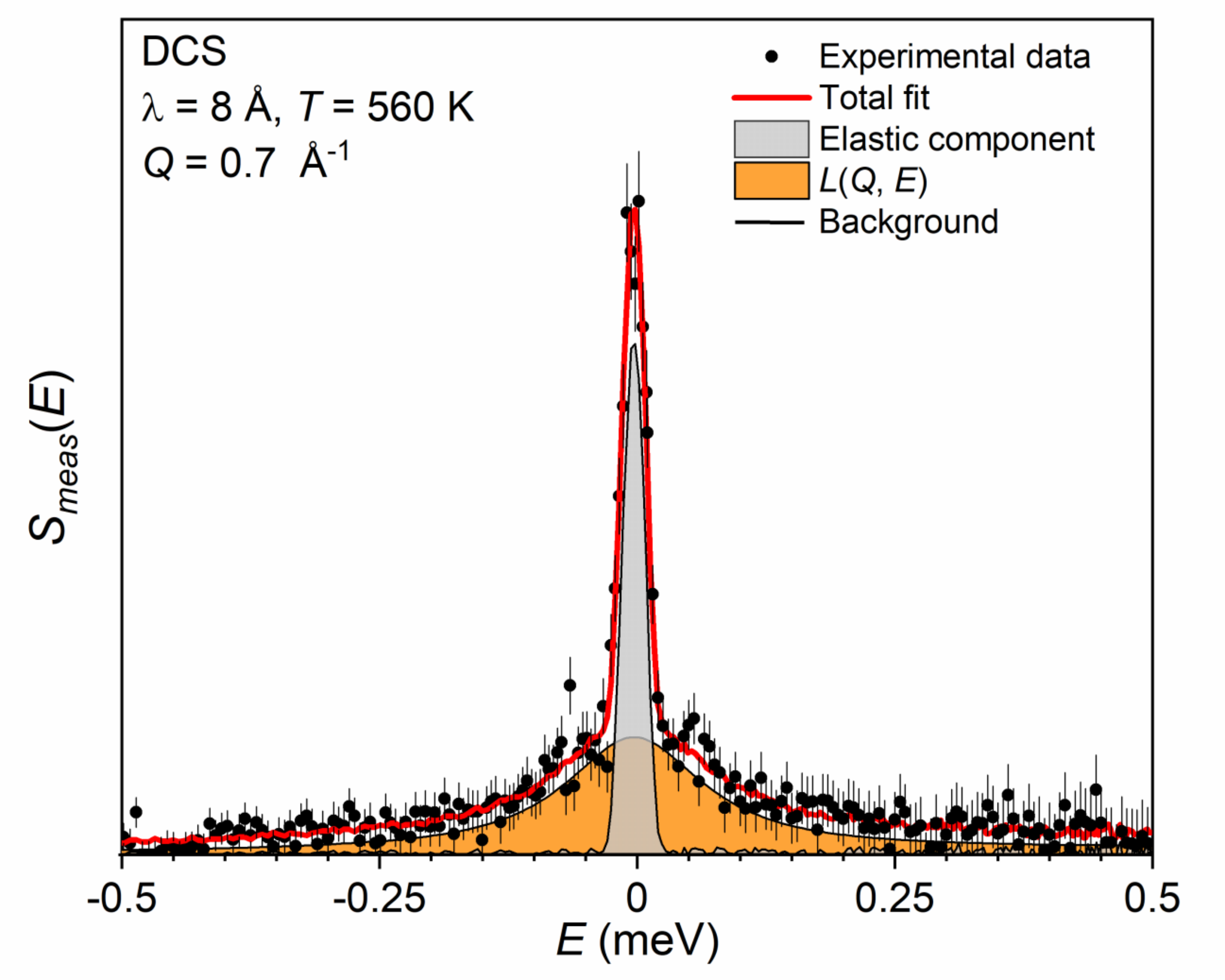

Figure S10. Exemplary QENS spectrum for $Cs_{0.93}Rb_{0.07}CB_{11}H_{12}$ at 560 K (Q = 0.7 Å^{-1}) using 8 Å wavelength neutrons (with 30 μeV fwhm resolution). The spectrum was fit with a delta function and one Lorentzian component L, both convoluted with the instrumental resolution function, on top of a flat background. The jump correlation frequency τ^{-1} is extracted from the Lorentzian component via the relation $\tau^{-1} = \Gamma/(2\hbar)$, where Γ is the Lorentzian fwhm linewidth and $\hbar$ is the reduced Planck's constant.

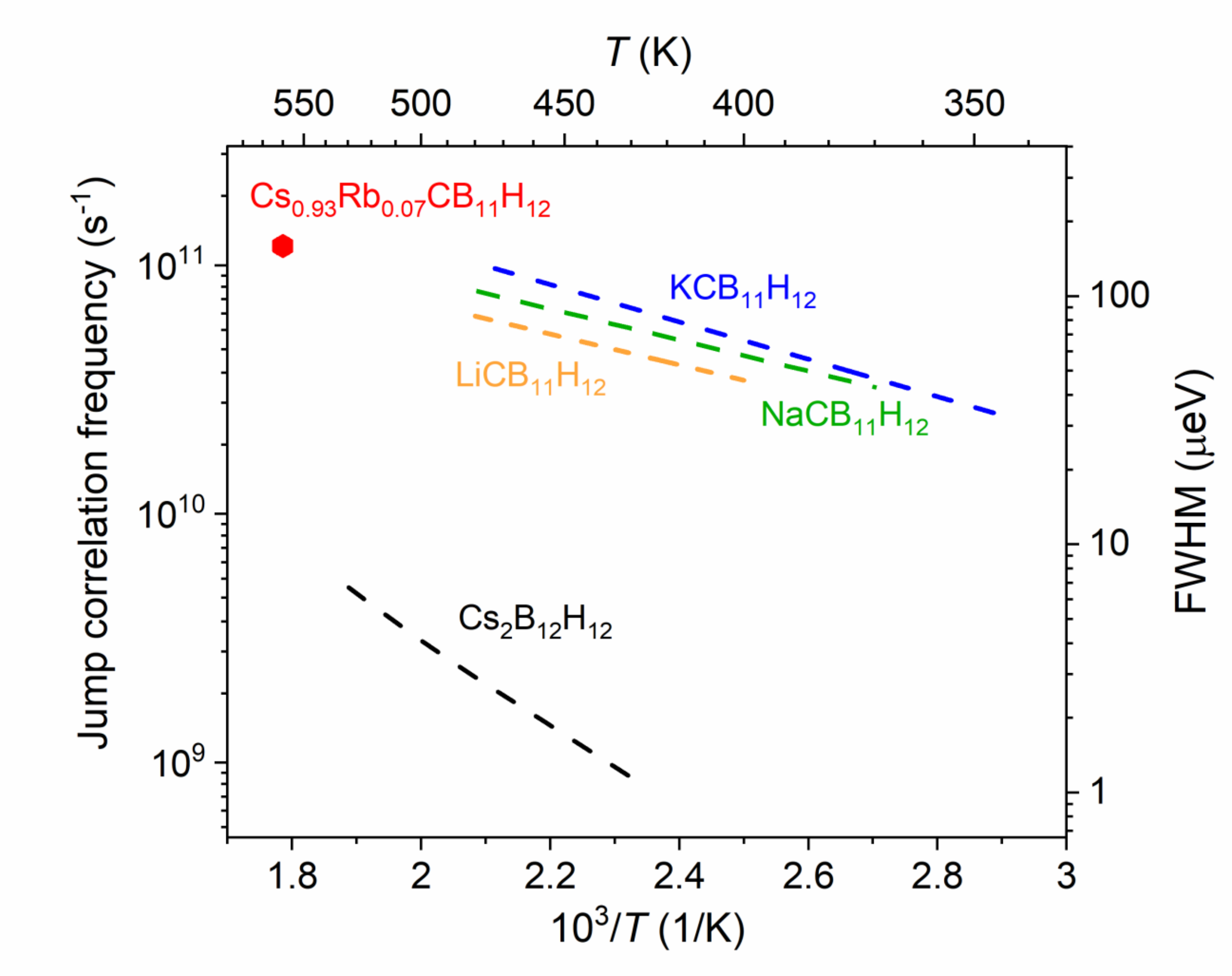

Figure S11. An Arrhenius plot (τ^{-1} vs. $1/T$) showing the 560 K anion jump correlation frequency [$1.19(9)\times10^{11}$ jumps/s^{-1}] for $Cs_{0.93}Rb_{0.07}CB_{11}H_{12}$ in relation to the temperature-dependent τ^{-1} behaviors (dashed lines) previously reported for $MCB_{11}H_{12}$ (M=Li, Na, and K) [7,16] and $Cs_2B_{12}H_{12}$ [18].

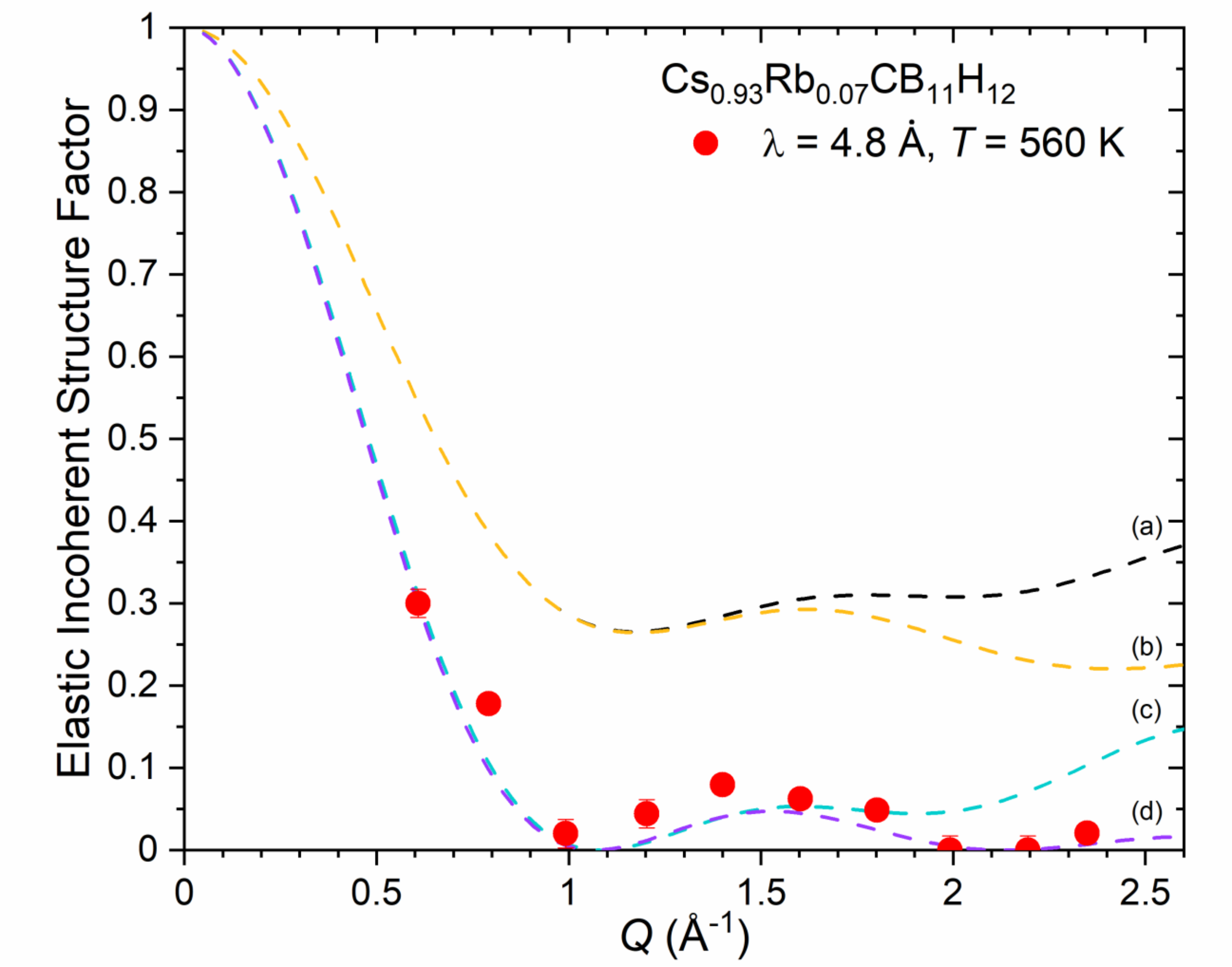

Figure S12. Elastic incoherent structure factor (EISF) data *vs*. *Q* for $Cs_{0.93}Rb_{0.07}CB_{11}H_{12}$ at 560 K derived from QENS measurements using 4.8 Å incident wavelength neutrons. EISF model curves for various anion reorientational mechanisms are shown for comparison: (a) uniaxial five-fold ($2\pi/5$) jumps around the C_5 anion symmetry axis, (b) uniaxial rotational diffusion around the C_5 anion symmetry axis (c) three-dimensional icosahedral tumbling among the twelve apical positions, and (d) isotropic rotational diffusion. Mathematical descriptions of the various model curves can be found in refs. [7,18]. The data are in line with a multi-directional reorientational mechanism, and the EISF values above ~1.8 Å^{-1} further indicate that the anions are taking small-angle jumps that more closely mimic isotropic rotational diffusion (curve d) rather than more discrete larger-angle icosohedral tumbling motions (curve c).

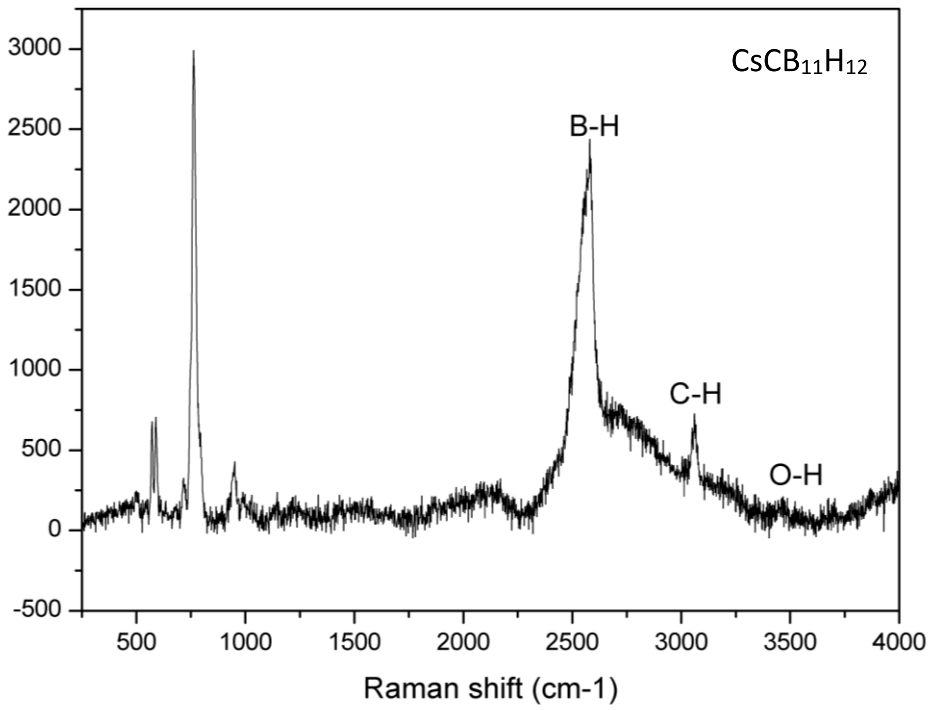

Figure S13: Raman spectrum of $CsCB_{11}H_{12}$ measured at room temperature for the same sample (in the same capillary) as used for SR-XPD experiments.

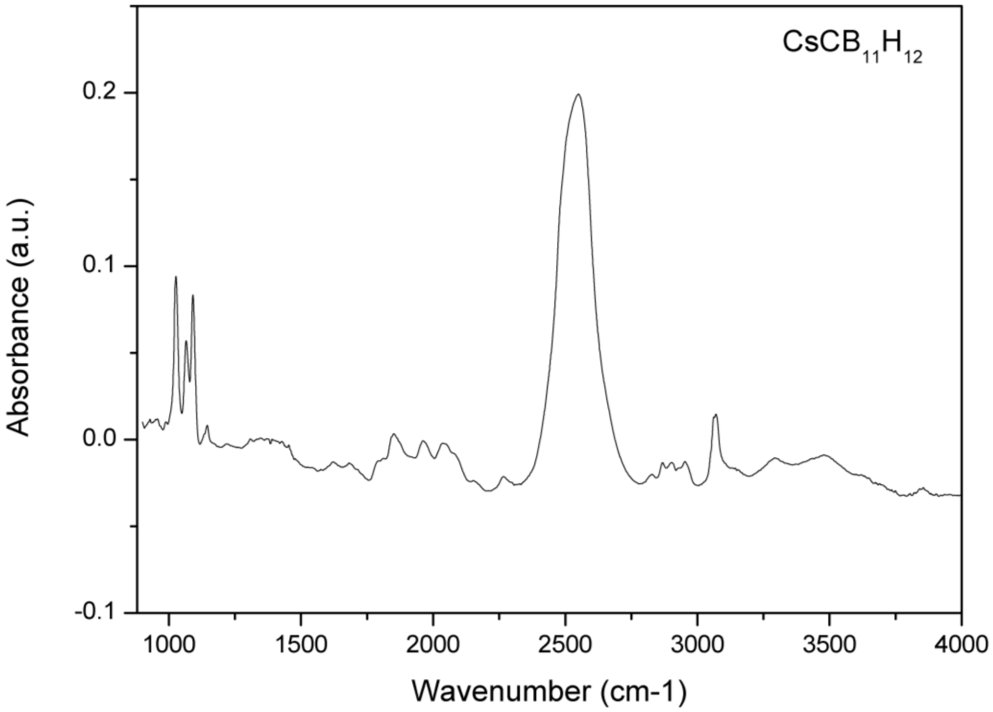

Figure S14: IR spectrum of $CsCB_{11}H_{12}$ measured at room temperature for the same sample as used for SR-XPD experiments.

We note that, for all supporting figures above, standard uncertainties are commensurate with the observed scatter in the data, if not explicitly designated by vertical error bars.
